# Age-Related Differences in Joint Kinematics and Spatiotemporal Parameters During Ramp Walking and Level Walking: Cross-Sectional Study

**DOI:** 10.2196/86036

**Published:** 2026-04-29

**Authors:** Daiki Shimotori, Soshi Fujisawa, Masahiro Nishimura, Tatsuya Yoshimi, Kenji Kato

**Affiliations:** 1 Laboratory of Practical Technology in Community, Assistive Robot Center, National Center for Geriatrics and Gerontology, Obu, Japan; 2 Laboratory of Clinical Evaluation with Robotics, Assistive Robot Center, National Center for Geriatrics and Gerontology, 7-430 Morioka-cho, Obu, 474-8511, Japan, 81 562 46 2311 ext 6112, 81 562 48 2373; 3 Department of Mechatronics Engineering, Faculty of Science and Technology, Meijo University, 1-501 Shiogamaguchi, Tempaku-kuNagoya, Aichi, 468-8502, Japan, 81-52-832-2566

**Keywords:** gait analysis, aging, ramp walking, markerless motion capture, living laboratory

## Abstract

**Background:**

Gait function is essential for mobility and independence in older adults. Although age-related gait decline on level walking has been studied extensively, there is less information on how aging affects gait during ramp walking, despite its relevance in daily life. Different biomechanical strategies are used during ramp ascent and descent; however, detailed joint kinematics remain unclear, particularly under real-world conditions.

**Objective:**

This study aims to investigate age-related differences in lower-limb joint angles and spatiotemporal parameters between level and ramp walking.

**Methods:**

Gait was assessed in 20 young (mean 31.3, SD 8.9 y) and 20 older (mean 64.2, SD 0.8 y) healthy adults using a markerless motion-capture system (Theia3D) in a living laboratory setting. Participants completed gait trials during level walking and on a 7° ramp (ascent and descent). Between-group comparisons of spatiotemporal parameters were performed using independent 2-tailed *t* tests, while joint angle data were analyzed using a linear mixed model with walking velocity as a covariate. Estimated marginal means were compared across age groups within each walking condition, and all *P* values were adjusted using the false discovery rate method. Effect sizes were calculated using Cohen *d* to examine whether between-group differences were more pronounced during ramp walking compared to level walking.

**Results:**

Ramp walking revealed multijoint kinematic alterations, while level walking showed limited differences (hip flexion and knee extension angles only). During ramp ascent, significant increases in knee flexion angle and reductions in ankle plantarflexion angle were observed. During ramp descent, multijoint changes were evident, including increased hip flexion and reduced knee extension and ankle plantarflexion angles. Effect sizes were particularly large during ramp walking (eg, knee flexion during ascent: |*d*|=2.37; knee extension during descent: |*d*|=1.87), while level walking showed large effect sizes for several parameters (eg, knee MaxExt_Stance_: |*d*|=1.18; hip MaxFlex_Stance_: |*d*|=1.17) despite reaching statistical significance for only a few parameters.

**Conclusions:**

In this cross-sectional sample, age-group differences in joint kinematics were more pronounced during ramp walking than during level walking, even after adjusting for walking velocity. This suggests that ramp walking may be a more sensitive task for detecting age-related adaptations compared to level walking. Markerless motion capture enables practical assessment in real-world settings. However, longitudinal studies are needed to determine whether these patterns predict functional decline.

## Introduction

Gait is fundamental to human mobility and independence. It is a daily activity performed without conscious effort. Despite this apparent ease, walking is a complex motor task that requires sophisticated coordination among the nervous, musculoskeletal, and cardiorespiratory systems to precisely control gait parameters (eg, walking velocity, cadence, and step length) throughout each gait cycle [1]. Consequently, gait function declines when these physiological systems are compromised [1]. Aging is one of the primary contributors; as the global population ages [2], more older adults experience declines in gait function [3]. This deterioration restricts essential activities of daily living—such as level walking, ramp walking, and stair climbing—and reduces physical activity, creating a negative cycle that accelerates motor function decline. Understanding the mechanisms underlying age-related gait decline is crucial for preventing this cycle and supporting older adults’ independence.

Extensive research has examined age-related gait decline, especially during level walking. Compared to young adults, older adults exhibit shorter stride lengths, reduced gait velocity [4], greater gait variability [5,6], prolonged double support time [7,8], and reduced ankle range of motion (RoM) [9]. Meta-analyses confirmed that ankle plantarflexion limitations were particularly pronounced in older populations (≥65 y), with reductions of approximately 5° compared to young adults [10]. These variations are attributed to age-related decline in neuromuscular function [11,12], including alterations in joint power generation and redistribution strategies [8,13]. Additionally, older adults consume 20% to 30% more physiological metabolic energy during walking than young adults [7], suggesting lower gait energy efficiency.

Unlike level walking, ramp walking involves distinct movement strategies [14,15], suggesting that age-related gait deterioration may vary by environment. Despite its importance in daily life, studies on age-related changes during ramp ascent and descent remain limited. Existing evidence suggests that older adults exhibit shorter stride length and reduced maximum ankle plantarflexion angle during ramp ascent [16] and reduced velocity, shorter stride length, and increased cadence during ramp descent on a 20% slope [17]. However, the underlying biomechanical mechanisms, such as joint kinematics, remain unclear. Notably, the reported reduction in ankle plantarflexion during ramp ascent [16] was based on treadmill assessment, which may not accurately reflect real-world conditions. Treadmill walking patterns have been shown to differ from natural gait on both level surfaces [18,19] and inclines [20]. This knowledge gap highlights the need for assessing ramp walking kinematics in settings that closely approximate real-world conditions.

To address this gap, our center established a “living laboratory” [21] to evaluate the activities of daily living of older adults under conditions that closely mimic real-world environments. The living laboratory features an indoor space and a separate outdoor recreational space simulating daily living environments, both equipped with a markerless motion-capture system, Theia3D (ver2021.2.0.1675, Theia Markerless Inc). Theia3D’s accuracy is comparable to conventional marker–based systems [22,23], with measurement errors below 5° [24], enabling natural gait assessments without special clothing or physical markers. This level of accuracy is sufficient for clinical applications [25]. Consequently, gait measurements performed in the living laboratory environment can provide accurate and reproducible data that reflect natural walking behavior [24].

This study assessed sagittal-plane lower-extremity joint angles in young and older adults across three living laboratory walking conditions: level walking, ramp ascent, and ramp descent. We aimed to test the hypothesis that age-related lower-extremity joint angle limitations are more pronounced in ramp walking than in level walking. We compared joint kinematics and calculated effect sizes between age groups in all 3 conditions to evaluate age-related differences in joint angle parameters during ramp walking compared to level walking.

Understanding these age-related lower-extremity kinematic adaptations may inform targeted interventions for preserving mobility and maintaining physical independence in older adults. Ultimately, this knowledge could improve the quality of life in aging populations.

## Methods

### Measurement Environment

**Figure 1.**
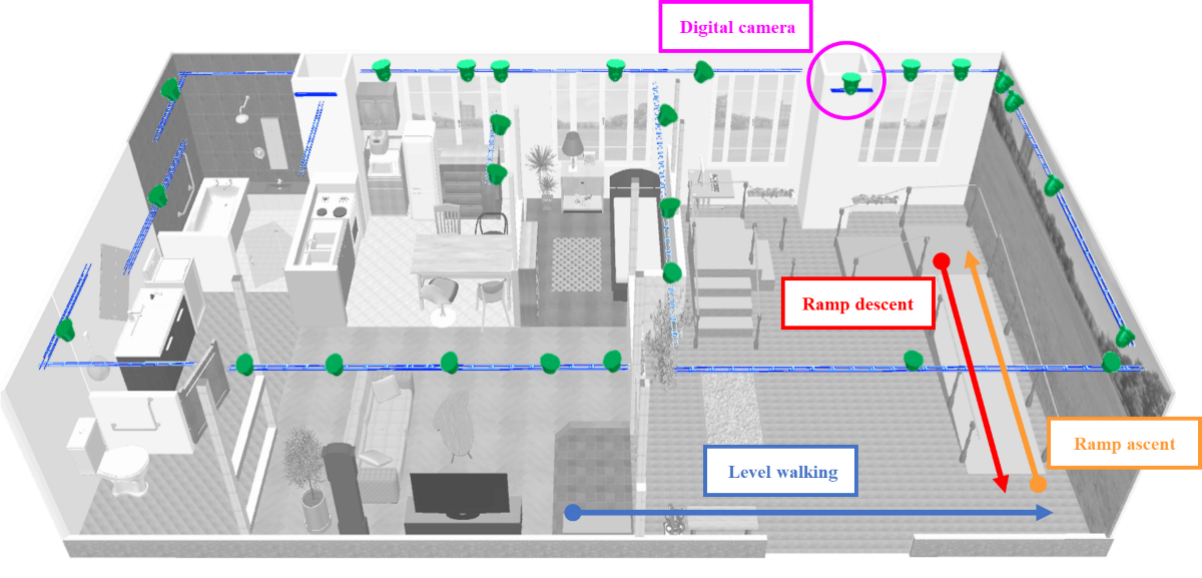
Experimental setup of the living laboratory and walkways for level walking and ramp walking measurements. Twenty-seven digital cameras for motion analysis are mounted on the ceiling and arranged to cover almost the entire area of the room (indicated by green objects).

This study was conducted in a living laboratory environment comprising outdoor and indoor spaces. Gait assessments were performed on level ground, and a ramp was used in the outdoor area. The level walking path was 7 m long, whereas the ramp was 3.25 m long, with a 7° incline and handrails on both sides (Figure 1). The slope angle used in this study was designed based on the maximum gradient specified in Japan’s Building Standards Act [26], which is commonly encountered in barrier-free public and residential environments. This design ensures that the experimental conditions reflect real-world walking scenarios.

### Participants

A total of 40 healthy participants were recruited, including 20 young adults (15 male adults, 5 female adults; mean age 31.3, SD 8.9 y; mean height 1.68, SD 0.09 m; mean weight 63.5, SD 12.4 kg) and 20 older adults (11 male adults, 9 female adults; mean age 64.2, SD 0.8 y; mean height 1.65, SD 0.08 m; mean weight 63.7, SD 10.5 kg). The younger adults were members of the rehabilitation staff at the National Center for Geriatrics and Gerontology. In contrast, the older adults were enrolled in the Silver Human Resources Center, a work-support organization in Japan. Participants were asked to wear comfortable clothing suitable for physical activity to the testing site. Participants with any neurological or musculoskeletal conditions affecting gait were excluded.

### Data Capture

Motion data were collected using 27 synchronized cameras (DSC-RX0M2; Sony Corporation) at a sampling rate of 60 Hz. The participants completed 5 trials each for 3 walking tasks performed in the following order: level walking, ramp ascent, and ramp descent (Figure 1). For each walking condition, the participants were positioned at designated starting points. The researcher confirmed that the participants were ready before signaling them to begin walking with the right foot in all trials. Measurements were stopped once the participants reached the designated end point and came to a complete stop. The participants were instructed to start walking at a self-selected speed and to refrain from using the handrails during the ramp task.

### Data Analysis

Video data were processed using Theia3D, which uses inverse kinematics and a 2-degrees-of-freedom knee model to estimate 3D body segment postures. A low-pass filter based on the generalized cross-validatory spline with a cutoff frequency of 6 Hz was applied. The calculated data were then exported to Visual3D (HAS-Motion) for spatiotemporal parameter analysis and kinematic analysis.

Initial contact (IC) and toe-off events were identified using the algorithm proposed by Zeni et al [27]. Visual confirmation and manual correction of misclassified events were performed using the 3D motion animation.

For level walking, data analysis began with the third step after the initial movement from a standing position and ended 3 steps before stopping. For ramp walking, data analysis began with the second step after the initial movement from a standing position and ended one step before stopping.

As previous studies have demonstrated that foot strike patterns influence joint kinematics [28], participants’ foot strike patterns were classified as heel strike or forefoot strike.

Spatiotemporal parameters, including cadence, velocity, and step length, were calculated for the analyzed gait cycles. These parameters were averaged across all measured trials within each walking condition, and the mean values were used as representative values for each participant.

Movements in the frontal and horizontal planes were excluded from the analysis to focus on sagittal plane joint angles. Sagittal angles of the bilateral hip, knee, and ankle joints were calculated as relative angles between the proximal and distal segments in their respective joint coordinate systems. For each trial, joint angles of the right and left limbs were time-normalized to represent 100% of the gait cycle, with 0% corresponding to IC and 100% to the next ipsilateral IC, and then averaged across limbs. The resulting waveforms were further averaged across all measured trials within each walking condition to obtain a representative waveform for each participant. Representative kinematic values were extracted based on the sagittal plane joint angle analysis conducted by Riazati et al [29] (Table 1).

**Table 1.** Definitions of representative values derived from time-series waveforms of sagittal-plane lower limb joint angles (hip, knee, and ankle).

Gait event	Description
ST1^a^	Stance phase 1, ipsilateral initial contact to mid-stance, representing the first half of the stance phase
ST2	Stance phase 2, mid-stance to ipsilateral toe-off, representing the second half of the stance phase
ST or SW^b^	Transition from stance-to-swing phase
RoM^c^	The entire range of motion over the full gait cycle, presented for hip, knee, and ankle joints in sagittal planes
MaxFlexStance	Maximum flexion angle (sagittal plane) achieved by the hip or knee joint during the stance phase
MaxFlexSwing	Maximum flexion angle (sagittal plane) achieved by the hip or knee joint during the swing phase
MaxFlexST1	Maximum flexion angle (sagittal plane) achieved by the knee joint during stance phase 1
MaxExtStance	Maximum extension angle (sagittal plane) achieved by the hip or knee joint during the stance phase
MaxDorsiflexionST2	Maximum dorsiflexion angle (sagittal plane) achieved by the ankle joint during the stance phase 2
MaxPlantarflexionST/SW	Maximum plantarflexion angle (sagittal plane) of the ankle joint at stance-to-swing transition
AnkleInitialContact	Ankle joint angle (sagittal plane) at initial contact

a ST: stance.

b SW: swing.

c RoM: range of motion.

### Statistical Analysis

For spatiotemporal parameters, data normality was assessed using the Shapiro-Wilk test, which confirmed normality for all parameters (*P*>.05). Mean values and SDs were calculated for cadence, velocity, and step length for each walking condition (level walking, ramp ascent, and ramp descent). Between-group comparisons were performed using independent 2-sample independent 2-tailed *t* tests. The false discovery rate (FDR) method was applied for multiple comparison correction. Effect sizes were calculated using Cohen *d* and interpreted as follows: negligible (|*d*|<0.2), small (|*d*|<0.5), medium (|*d*|<0.8), and large (0.8≤|*d*|). The purpose of calculating effect sizes was to determine whether between-group differences were more pronounced during ramp ascent or ramp descent walking compared to level walking.

For joint angle data, representative values (eg, RoM, MaxFlex_Stance_, MaxFlex_Swing_) were extracted for the hip, knee, and ankle joints for each participant and walking condition, resulting in a total of 36 joint angle parameters for analysis. A linear mixed model (LMM) was used to adjust for the confounding effect of walking velocity, which differed significantly between age groups and is known to influence joint kinematics [30]. The model was configured with joint angle as the dependent variable, walking velocity as a covariate, age group (young adults vs older adults) as a between-participant factor, and walking condition (level walking, ramp ascent, ramp descent) as a within-participant factor. Model fitting was performed using the lme4 package (version 1.1.37) [31], with participant included as a random intercept to account for repeated measures within individuals. Representative kinematic values from each participant and walking condition were included as observations.

The normality of residuals from the LMM was assessed using the Shapiro-Wilk test. The results indicated a deviation from normality for the maximum ankle plantarflexion angle (*P*=.04). However, the visual inspection of the Q-Q plots revealed no extreme outliers, suggesting that the deviation was minor. Given these findings and the robustness of LMMs to minor normality violations [32], the maximum ankle plantarflexion angle was considered to meet the normality assumption.

Main effects and interactions were tested using type 3 analysis of variance with Satterthwaite approximation for degrees of freedom, implemented via the lmerTest package (version 3.1.3) [33]. For joint angle parameters showing significant interactions, simple main effects were evaluated using the emmeans package (version 1.11.2‐8) [34], which calculated estimated marginal means and pairwise comparisons between age groups within each walking condition. The FDR method was applied to all *P* values obtained from the simple main effects tests to correct for multiple comparisons. Effect sizes (Cohen *d*) were calculated as the difference in estimated marginal means divided by the residual standard deviation from the LMM and interpreted using the same criteria as those for the spatiotemporal parameters.

All statistical analyses were performed using R (version 4.4.2) and RStudio (version 2025.09.2 Build 418), with a significance level set at *P*<.05.

### Ethical Considerations

The study protocols were approved by the Ethics and Conflict of Interest Committee of the National Center for Geriatrics and Gerontology (approval numbers 1636 and 1637). Written informed consent was obtained from all participants prior to their inclusion in the study. All participants were assigned unique identification codes, and personal identifiers were removed from the dataset to ensure anonymity. The younger adult participants (rehabilitation staff) did not receive compensation. The older adult participants, recruited through the Silver Human Resources Center, received monetary compensation.

## Results

### Foot-Strike Pattern and Spatiotemporal Parameters

An analysis of foot-strike patterns revealed that all the lower limbs analyzed showed a heel-strike pattern. Table 2 presents the results of the spatiotemporal parameters, including the cadence (steps/min), velocity (m/s), and step length (m).

**Table 2.** Summary of spatiotemporal gait parameters (cadence [steps/min], velocity [m/s], and step length [m]) during level walking and ramp ascent or descent.

Task and spatiotemporal parameters	Young adults, mean (SD)	Older adults, mean (SD)	*P* value^a^	Effect sizes^b^	Effect size magnitude
Cadence (step/min)					
Level walking	114.14 (5.43)	114.13 (8.30)	>.99	0.001	Negligible
Ramp ascent	104.16 (5.70)	102.04 (7.74)	.40	0.312	Small
Ramp descent	112.60 (5.35)	112.04 (10.60)	.60	0.067	Negligible
Velocity (m/s)					
Level walking	1.39 (0.13)	1.28 (0.18)	.06	0.684	Medium
Ramp ascent	1.24 (0.10)	1.08 (0.10)	*<.001*	1.614	Large
Ramp descent	1.27 (0.15)	1.11 (0.16)	.004	1.055	Large
Step length (m)					
Level walking	0.73 (0.06)	0.67 (0.07)	*.01*	0.967	Large
Ramp ascent	0.72 (0.05)	0.64 (0.05)	*.003*	1.554	Large
Ramp descent	0.67 (0.07)	0.59 (0.07)	*.003*	1.077	Large

a Values in italics indicate statistically significant differences at *P*<.05.

b Effect sizes were calculated using Cohen *d* and interpreted as negligible (|*d*|<0.2), small (|*d*|<0.5), medium (|*d*|<0.8), or large (0.8 ≤ |*d*|).

Differences were observed in the velocity and step length, with the older adults group exhibiting a lower velocity and smaller step length than the young adults group; however, there were no differences in cadence. Specifically, the mean walking velocity was numerically lower in the older group than in the young group during level walking (young adults: 1.39, SD 0.13 m/s vs older adults: 1.28, SD 0.18 m/s; *P*=.06), though this difference did not reach statistical significance. The velocity difference was significant during ramp ascent (young adults: 1.24, SD 0.10 m/s vs older adults: 1.08, SD 0.10 m/s; *P*<.001) and ramp descent (young adults: 1.27, SD 0.15 m/s vs older adults: 1.11, SD 0.16 m/s; *P*=.004).

Although no significant difference in height was observed between the young adult and older adult groups (independent 2-tailed *t* test; *P*=.25), the mean step length showed significant differences under all conditions, with older adults exhibiting shorter step length than young adults: level walking (young adults: 0.73, SD 0.06 m vs older adults: 0.67, SD 0.07 m; *P*=.01), ramp ascent (young adults: 0.72, SD 0.05 m vs older adults: 0.64, SD 0.05 m; *P*=.003), and ramp descent (young adults: 0.67, SD 0.07 m vs older adults: 0.59, SD 0.07 m; *P*=.003).

### Typical Value of Joint Angle

**Figure 2.**
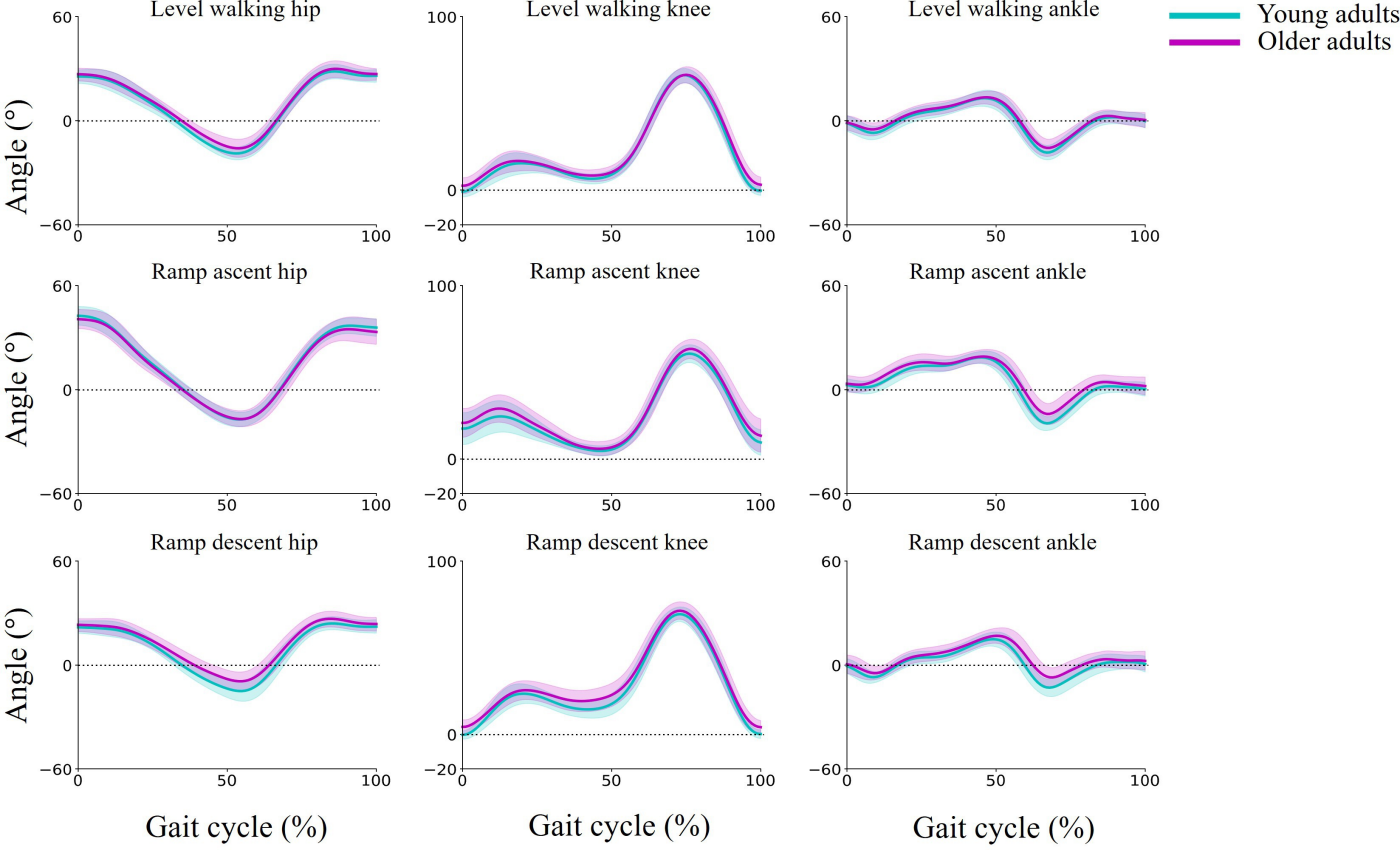
Time-series waveforms of lower limb joint angles (hip, knee, and ankle) in the sagittal plane during level walking and ramp ascent/descent. The waveforms are normalized with one gait cycle defined as 100%. The shaded areas around the waveforms represent the standard deviation.

Figure 2 shows the time-series waveforms of the hip, knee, and ankle joint angles throughout the gait cycle for both age groups. Table 3 summarizes the representative values derived from these waveforms.

During level walking, significant differences were observed between age groups for 2 parameters: hip MaxFlex_Stance_ (young adults: 24.1° vs older adults: 26.7°; *P*=.04) and knee MaxExt_Stance_ (young adults: 1.4° vs older adults: −1.5°; *P*=.009).

During ramp ascent, significant differences between age groups were observed for 4 parameters: hip MaxExt_Stance_ (young adults: 17.3° vs older adults: 20.4°; *P*=.04), knee MaxFlex_ST1_ (young adults: 25.0° vs older adults: 33.4°; *P*<.001), ankle RoM (young adults: 39.9° vs older adults: 35.8°; *P*=.009), and ankle MaxPlantarflexion_ST/SW_ (young adults: 20.5° vs older adults: 15.4°; *P*<.001).

During ramp descent, significant differences between age groups were observed for 5 parameters: hip MaxFlex_Stance_ (young adults: 21.9° vs older adults: 25.7°; *P*=.008), hip MaxFlex_Swing_ (young adults: 24.8° vs older adults: 28.2°; *P*=.018), knee MaxFlex_ST1_ (young adults: 23.5° vs older adults: 28.7°; *P*=.018), knee MaxExt_Stance_ (young adults: 0.5° vs older adults: −4.1°; *P*<.001), and ankle MaxPlantarflexion_ST/SW_ (young adults: 13.8° vs older adults: 9.9°; *P*=.012).

Regarding effect sizes, large effect sizes were observed during level walking for hip MaxFlex_Stance_ (|*d*|=1.17), hip MaxFlex_Swing_ (|*d*|=1.16), knee RoM (|*d*|=0.80), knee MaxFlexST1 (|*d*|=1.00), knee MaxExt_Stance_ (|*d*|=1.18), ankle RoM (|*d*|=0.86), and ankle MaxPlantarflexion_ST/SW_ (|*d*|=0.83). A small effect size was observed for knee MaxFlex_Swing_ (|*d*|=0.22). Negligible effect sizes were observed for hip MaxExt_Stance_ (|*d*|=0.02) and ankle MaxDorsiflexion_ST2_ (|*d*|=0.00).

For ramp ascent, large effect sizes were observed for hip MaxExt_Stance_ (|*d*|=1.36), knee RoM (|*d*|=0.82), knee MaxFlex_ST1_ (|*d*|=2.37), knee MaxFlex_Swing_ (|*d*|=1.73), ankle RoM (|*d*|=1.91), and ankle MaxPlantarflexion_ST/SW_ (|*d*|=2.35). A medium effect size was observed for hip MaxFlex_Stance_ (|*d*|=0.50), and small effect sizes were observed for hip MaxFlex_Swing_ (|*d*|=0.21), knee MaxExt_Stance_ (|*d*|=0.49), and ankle MaxDorsiflexion_ST2_ (|*d*|=0.44).

For ramp descent, large effect sizes were observed for hip MaxFlex_Stance_ (|*d*|=1.67), hip MaxFlex_Swing_ (|*d*|=1.67), hip MaxExt_Stance_ (|*d*|=1.02), knee MaxFlex_ST1_ (|*d*|=1.46), knee MaxFlex_Swing_ (|*d*|=1.38), knee MaxExt_Stance_ (|*d*|=1.87), ankle RoM (|*d*|=0.88), ankle MaxDorsiflexion_ST2_ (|*d*|=1.18), and ankle MaxPlantarflexion_ST/SW_ (|*d*|=1.79). A medium effect size was observed for knee RoM (|*d*|=0.52).

**Table 3.** Representative joint angle results (hip, knee, and ankle) in the sagittal plane during level walking and ramp ascent/descent.

Task and joint parameters	Young adults, EMMs^a^	Older adults, EMMs	*P* value^b^	Effect sizes^c^	Effect size magnitude
Level walking					
Hip (°)					
RoM^d^	44.5	47.2	n.s.^e^	—^f^	—
MaxFlex_Stance_	24.1	26.7	*.04*	1.17	Large
MaxFlex_Swing_	27.9	30.2	.09	1.16	Large
MaxExt_Stance_	16.2	16.2	.995	0.02	Negligible
Knee (°)					
RoM	68.1	65.6	.19	0.80	Large
MaxFlex_ST1_	13.7	17.3	.09	1.00	Large
MaxFlex_Swing_	66.2	66.6	.81	0.22	Small
MaxExt_Stance_	1.4	−1.5	*.009*	1.18	Large
Ankle (°)					
RoM	32.6	30.7	.19	0.86	Large
MaxDorsiflexion_ST2_	14.1	14.1	.995	0.00	Negligible
MaxPlantarflexion_ST/SW_	18.5	16.7	.19	0.83	Large
InitialContact	−2.0	−0.6	n.s.	—	—
Ramp ascent					
Hip (°)					
RoM	60.1	63.4	n.s.	—	—
MaxFlex_Stance_	42.6	43.7	.39	0.50	Medium
MaxFlex_Swing_	38.9	39.3	.79	0.21	Small
MaxExt_Stance_	17.3	20.4	*.04*	1.36	Large
Knee (°)					
RoM	58.0	60.5	.19	0.82	Large
MaxFlex_ST1_	25.0	33.4	*<.001*	2.37	Large
MaxFlex_Swing_	61.0	64.3	.05	1.73	Large
MaxExt_Stance_	−4.0	−5.2	.27	0.49	Small
Ankle (°)					
RoM	39.9	35.8	*.009*	1.91	Large
MaxDorsiflexion_ST2_	19.3	20.0	.55	0.44	Small
MaxPlantarflexion_ST/SW_	20.5	15.4	*<.001*	2.35	Large
InitialContact	1.3	3.8	n.s.	—	—
Ramp descent					
Hip (°)					
RoM	39.6	41.2	n.s.	—	—
MaxFlex_Stance_	21.9	25.7	*.008*	1.67	Large
MaxFlex_Swing_	24.8	28.2	*.02*	1.67	Large
MaxExt_Stance_	14.8	12.5	.11	1.02	Large
Knee (°)					
RoM	71.0	69.4	.39	0.52	Medium
MaxFlex_ST1_	23.5	28.7	.*02*	1.46	Large
MaxFlex_Swing_	69.9	72.6	.11	1.38	Large
MaxExt_Stance_	0.5	−4.1	<.001	1.87	Large
Ankle (°)					
RoM	29.2	27.3	.19	0.88	Large
MaxDorsiflexion_ST2_	15.3	17.4	*.11*	1.18	Large
MaxPlantarflexion_ST/SW_	13.8	9.9	.01	1.79	Large
InitialContact	−0.3	2.2	n.s.	—	—

a EMM: estimated marginal mean.

b Values in italics indicate statistically significant differences at *P*<.05.

c Effect sizes are reported as Cohen *d* and interpreted as negligible (|*d*|<0.2), small (0.2 ≤ |*d*|<0.5), medium (0.5 ≤ |*d*|<0.8), or large (0.8 ≤ |*d*|).

d RoM: range of motion.

e n.s.: For parameters with no significant interaction (age group×task condition), simple main effects were not evaluated.

f Effect sizes were not calculated,

## Discussion

### Principal Findings

This study demonstrated that, after statistically adjusting for walking velocity using the LMM, ramp walking showed larger age-group differences in lower limb joint kinematics compared to level walking. While level walking showed large effect sizes for several parameters (|*d*|=0.80‐1.18), only 2 parameters demonstrated statistical significance after adjusting for multiple comparisons using the FDR. In contrast, ramp conditions revealed larger effect sizes with high statistical significance. During ramp ascent, 4 parameters showed significant differences with large effect sizes (|*d*|=1.36‐2.37), with the largest effects observed in knee flexion during early stance (|*d*|=2.37) and ankle plantarflexion (|*d*|=2.35). During ramp descent, 5 parameters showed significant differences with large effect sizes (|*d*|=1.46‐1.87). The persistence of these large effect sizes after controlling for walking velocity differences indicates that the observed kinematic differences reflect age-related motor adaptations that emerge under challenging mobility conditions rather than simply being artifacts associated with different walking velocities. From these findings, it can be inferred that ramp walking has the potential to assess age-related kinematic adaptations that were less pronounced during level walking.

### Physiological Mechanisms and Gait Strategy Adaptation

Age-related reductions in ankle plantarflexion angles during level walking have been consistently reported in previous studies examining older populations (≥65 y). A systematic review and meta-analysis by Pol et al [10] reported that older adults (≥65 y) showed significantly lower maximum ankle plantarflexion angles than young adults during level walking (weighted mean difference: −5.15°). In our study, although older adults tended to show lower maximum ankle plantarflexion angles than young adults during level walking (young adults: 18.5° vs older adults: 16.7°, |*d*|=0.83), this parameter did not reach significance, possibly reflecting the younger age of our cohort (mean: 64.2 y). While significant differences were observed in hip flexion and knee extension during stance, several parameters exhibited large effect sizes (|*d*|=0.80‐1.18) without reaching significance, suggesting that level walking may not fully reveal age-related motor adaptations.

During ramp ascent walking, older adults showed coordinated multijoint adaptations. Significantly reduced ankle plantarflexion (young adults: 20.5° vs older adults: 15.4°, |*d*|=2.35) and ankle range of motion (young adults: 39.9° vs older adults: 35.8°, |*d*|=1.91) indicate compromised push-off capacity, consistent with age-related reductions in ankle power generation observed during level walking [8] and altered triceps surae muscle properties [35,36]. Hip extension during terminal stance significantly increased (young adults: 17.3° vs older adults: 20.4°, |*d*|=1.36), demonstrating compensatory reliance on proximal joints—a redistribution pattern where older adults shift from ankle-dominant to hip-dominant power generation [13], which becomes particularly pronounced during inclined walking [16].

In addition, knee flexion during early stance markedly increased (young adults: 25.0° vs older adults: 33.4°, |*d*|=2.37), the largest effect observed. This likely reflects reduced step length associated with diminished propulsive capacity, whereby ground contact occurs before full knee extension. This pattern may also represent an adaptive strategy for stability during upward ambulation. These coordinated adaptations across the ankle, knee, and hip joints underscore the integrated nature of age-related motor modifications during inclined walking.

Furthermore, multijoint adaptations were also observed in older adults during ramp descent. During descent, significant increases in hip MaxFlex_Stance_ (young adults: 21.9° vs older adults: 25.7°), hip MaxFlex_Swing_ (young adults: 24.8° vs older adults: 28.2°), and knee MaxFlex_ST1_ (young adults: 23.5° vs older adults: 28.7°) angles were observed, along with significant reductions in knee MaxExt_Stance_ (young adults: 0.5° vs older adults: −4.1°) and ankle MaxPlantarflexion_ST/SW_ (young adults: 13.8° vs older adults: 9.9°) angles.

These coordinated adaptations can be understood through 2 complementary biomechanical perspectives. First, from a neuromuscular control standpoint, ramp descent requires greater eccentric muscle contractions compared to level walking [37], particularly increasing knee and ankle joint angles [38]. Age-related decline in eccentric contraction capacity compromises postural control ability and increases fall risk [39]. The observed reductions in knee extension and ankle plantarflexion range of motion in older adults may therefore reflect declining eccentric control capacity during controlled descent.

Second, from a mechanical perspective, ramp descent walking intensifies the demand for gravitational impact absorption. Older adults commonly exhibit reduced ankle range of motion, particularly in plantarflexion [10], which alters propulsion, shock absorption, and kinetic patterns during gait [40]. During descent, the reduced knee extension and ankle plantarflexion angles observed in our older participants may indicate a diminished capacity to generate the negative joint torques required for effective shock absorption, potentially increasing mechanical loading on the lower limbs.

These coordinated joint restrictions likely represent adaptive strategies for maintaining postural stability during the challenging task of controlled lowering against gravity [14,15].

### Clinical Significance

Most clinical gait evaluations rely on level walking as the standard assessment [7]. However, our findings reveal that ramp walking reveals age-related kinematic adaptations that level walking assessments largely miss. After controlling for walking velocity, the substantially larger differences during ramp conditions (|*d*|=1.46‐2.37 vs 0.80‐1.18) demonstrate systematic motor adaptations that may warrant clinical attention. These patterns—particularly the large effect sizes in ankle plantarflexion during ascent (|*d*|=2.35) and multijoint coordination during descent—represent specific targets for intervention development. Although our cross-sectional design cannot establish predictive relationships for functional decline, the magnitude and consistency of these differences suggest clinical relevance. Markerless motion capture technology makes such assessments practically feasible [41], enabling longitudinal validation studies to examine whether these patterns predict subsequent mobility decline or fall incidence over 5‐ to 10-year follow-up periods. However, it should be noted that our research focused on identifying group-level kinematic differences and did not include diagnostic accuracy analyses. Further research, including longitudinal validation followed by diagnostic studies, is required before these kinematic patterns can be applied for clinical screening purposes.

### Future Perspectives for Living Laboratory

The living laboratory’s naturalistic outdoor setting with fixed inclines and markerless motion capture differs fundamentally from treadmill-based protocols used in previous ramp walking studies [16,18-20]. Treadmill constraints—predetermined speeds, continuous belt motion, and laboratory boundaries—may mask natural motor adaptations. Our environment allowed spontaneous ramp walking without such constraints, potentially capturing motor patterns that more closely resemble daily-life behavior compared to laboratory-constrained assessments. This ecological validity may partially explain the substantial age-related differences we observed. Furthermore, the living laboratory serves as a safe and iterative testing ground for emerging assistive technologies before clinical deployment. For example, the biomechanics-derived “Hug” transfer-assist robot was iteratively refined based on user feedback to better meet user requirements [42]. Similarly, the observed age-related limitations in ankle plantarflexion during ramp ascent could inform the development and testing of targeted interventions, such as mobility aids or training programs specifically designed to support inclined walking in older adults. Beyond these applications, the living laboratory may also be used to evaluate diverse technologies, including mobility aids, behavior-modifying sensor systems, and smart care robotics, and provide empirical data on their usability, safety, and functional effectiveness. Future research could leverage this infrastructure to incorporate more diverse real-world walking challenges, including stair negotiation, directional changes, and navigating uneven terrain or lateral slopes [43,44], thereby developing more comprehensive motor function assessment frameworks that better reflect the full spectrum of mobility demands faced by older adults in daily life. Through this infrastructure, the living laboratory facilitates the translation of research findings into practical interventions for supporting mobility in aging populations.

### Research Limitations and Future Directions

This study has several limitations. First, only kinematic data were analyzed; kinetic data (eg, joint moments or forces) were not analyzed. Fully understanding gait kinematic strategies requires examining joint angles and mechanical power distribution. Prior studies show that older adults rely more on proximal joints [13,16], suggesting age-related motor strategy reorganization. Future studies that combine markerless motion capture with ground reaction force measurements and inverse dynamics via musculoskeletal modeling can offer deeper insights into age-related motor coordination changes [45,46].

Second, this study has limitations related to its design and sample characteristics. The participants were relatively young older adults (mean age 64.2, SD 0.8 y), limiting generalizability to frail or older populations. Additionally, sex distribution was unbalanced between groups (young: 15 male adults, 5 female adults; older: 11 male adults, 9 female adults), and given the well-documented sex-related differences in gait biomechanics [47], this may have influenced the results. Furthermore, the cross-sectional design demonstrates age-group differences at a single time point but not longitudinal progression or predictive value for functional outcomes. Future studies should include broader age ranges and longitudinal designs to capture the full extent of age-related gait changes and determine whether these kinematic patterns predict subsequent falls or mobility impairment.

Third, cognitive and perceptual factors, such as fear of falling and attentional load, were not examined in this study despite their known impact on gait patterns. Research has demonstrated that cognitive-motor interactions, including dual-task performance, reveal age-related declines in attentional capacity and gait control during walking [48]. Future studies should incorporate assessments of these factors, including dual-task walking paradigms, to better understand the interplay between cognitive demands and motor control in older adults.

Fourth, while we statistically adjusted for walking velocity using linear mixed-effects models, several potential confounding factors (habitual physical activity, muscle strength, comorbidities, medications, footwear, and fear of falling) were not measured or controlled. Participants were recruited through a senior employment center, suggesting they were relatively healthy and physically active older adults. However, the lack of comprehensive health and physical function assessments limits our ability to determine whether the observed kinematic differences reflect aging per se or differences in health status and physical capabilities between volunteer groups. Future studies should include detailed assessments of these factors to better isolate age-related changes from health-related confounders.

Despite these limitations, this study demonstrates that ramp walking reveals greater age-related kinematic differences than level walking, even after controlling for walking velocity. Addressing these limitations in future research will advance the understanding of age-related gait mechanisms and their clinical relevance.

### Conclusion

This study demonstrated that ramp walking is more effective than level walking in revealing age-related differences in gait. After adjusting for the confounding factor related to walking velocity, ramp conditions showed substantially greater differences, suggesting that ramp walking may help assess possible age-related motor decline more acutely than level walking. However, since ours was a cross-sectional study, we cannot establish predictive value. Longitudinal studies are needed to determine whether these patterns predict functional decline. This study provides cross-sectional evidence of age-group differences in gait kinematics during ramp walking and level walking, with larger between-group differences observed during ramp conditions in this sample.

## Abbreviations

IC: initial contact
RoM: range of motion
LMM: linear mixed model
FDR: false discovery rate
